# I Phillips

**Published:** 1889-09

**Authors:** 


**I. Phillips.**—The Surgical Instrument man.—Read his advertisement of *Cut Rates* on page 17 of our advertising department. Physicians desiring instruments should communicate with this House. Those visiting the City should call on Phillips. He charges nothing for looking at his select and beautiful stock, and his polite and courteous reception of his customers makes a visit to his store very pleasant.

